# Adverse Events in Obstetrics: Impacts on Providers and Staff of Maternity Care

**DOI:** 10.7759/cureus.6732

**Published:** 2020-01-21

**Authors:** Samantha L Margulies, Joshua Benham, Joan Liebermann, Richard Amdur, Nancy Gaba, Jennifer Keller

**Affiliations:** 1 Obstetrics, Gynecology and Reproductive Sciences, Yale Medicine, New Haven, USA; 2 Obstetrics and Gynecology, Sutter West Bay Medical Group, San Francisco, USA; 3 Psychiatry and Behavioral Sciences; Obstetrics and Gynecology, The George Washington University School of Medicine and Health Sciences, Washington, D.C., USA; 4 Surgery, The George Washington University School of Medicine and Health Sciences, Washington, D.C., USA; 5 Obstetrics and Gynecology, The George Washington University School of Medicine and Health Sciences, Washington, D.C., USA

**Keywords:** adverse events, second victim, obstetric traumas, maternal mortality, perinatal outcomes, second victim syndrome, obstetrics/gynecology, obstetrics and gynecology

## Abstract

Objective

To determine the frequency of maternity health employee experiences with maternal and perinatal/neonatal adverse outcomes and gain a deeper understanding of how these experiences impact the providers.

Design

Single-institution observational study from 2016.

Setting

The George Washington University Hospital.

Population

Labor and delivery, postpartum, and neonatal intensive care staff.

Methods

An anonymous survey was distributed to maternity staff inquiring about feelings surrounding maternal and perinatal/neonatal adverse outcomes. Predictors included demographics and job-related variables. Associations were examined using univariable and multivariable analyses.

Main Outcome Measures

Outcomes included depression, post-traumatic stress disorder symptoms, and work-related problems following the event.

Results

A total of 105 employees of approximately 230 eligible employees answered the survey, including obstetrics and gynecology and anesthesia physicians (residents and attendings), midwives, nurses, nurse practitioners, and medical technicians with a response rate of 46%. Being a physician was protective against symptoms of depression and post-traumatic stress disorder symptoms. Resident physicians had higher levels of anxiety/depression compared to attendings. Statistically significant variables predictive of negative repercussions included non-physician status (p=.045), substance use (p=.0036), considering a career change (p<.0001) and seeking mental health treatment (p=.0005). About half of the respondents were aware that processes exist to help them cope with adverse outcomes.

Conclusions

Non-physicians, those using substances, those considering career change, and those seeking mental health treatment are more likely to experience anxiety/depression and post-traumatic stress symptoms after a maternal or perinatal/neonatal loss. These individuals should be identified and offered additional support.

## Introduction

In the United States, approximately 700 women die annually from pregnancy or delivery complications, and there are more than one million fetal losses annually [1-2]. In obstetrics and gynecology (OB/GYN), providers frequently care for women and families during such events.

Studies examining physician responses to the loss of a patient in specialties such as oncology, psychiatry, and pediatrics have been reported [3-7]. Some describe the “second victim” phenomenon as a situation in which a healthcare provider is involved in an unanticipated adverse patient event or medical error left feeling traumatized, responsible for the outcome or as if they failed the patient [8]. Minimal-to-no data exist examining the impact of a maternal/perinatal loss on providers of maternity care. Increased attention has been placed on provider wellness by the Accreditation Council on Graduate Medical Education (ACGME), American College of Obstetricians and Gynecologists (ACOG), and other stakeholders [9].

The purpose of this study is to determine the frequency of maternity health employee experiences with maternal and perinatal/neonatal adverse outcomes and to gain a deeper understanding of how these experiences impact the providers.

## Materials and methods

A questionnaire was developed and distributed in 2016 to those working regularly in the Women’s Services Department at The George Washington University Hospital (GWUH), an urban, academic hospital with an 18-bed, Level III Neonatal Intensive Care Unit (NICU). Survey participants included attending physicians (OB/GYN and anesthesia), residents, midwives, social workers, nurse practitioners, and hospital employees in foodservice and housekeeping.

The survey assessed symptoms of anxiety, depression, post-traumatic stress disorder (PTSD) symptoms, work-related problems such as interpersonal relationships at work and social support. The validated Patient Health Questionnaire-4 and the validated four-question Primary Care Post-Traumatic Stress Disorder Screen (PC-PTSD) were used to screen for symptoms of anxiety, depression, and PTSD symptoms, respectively. Responders were asked to recall their feelings immediately after the adverse event. The data were maintained using REDcap, a secure online data management system.

Approval from the Institutional Review Board at The George Washington University Office of Human Research was obtained. Participants gave informed consent prior to completing the survey.

Due to the small sample size, Cronbach’s alpha was used to group conceptually similar questionnaire items and similar predictor variables. Items that had Cronbach’s alpha >0.80 were considered to measure a single construct; their mean was used as the measure of that construct after standardizing to equally weight items, allowing condensation of similar items into four discrete scales: anxiety/depression, PTSD symptoms, work-related problems, and social support. Predictor variables of interest included demographics, job type, level of perceived support at work, and history of exposure to loss events at work and in one’s personal life. For the dependent variable in multivariable models, a global negative repercussion score as a second-order factor was created by taking the mean of the scores for anxiety/depression, PTSD symptoms, and work-related problems.

Variable distributions were examined for normality and outliers using histograms. Univariate associations of predictor variables with continuous outcome variables were tested using two-tailed, between-groups t-tests for binary predictors or analysis of variance for multilevel categorical predictors or Pearson correlation coefficient for continuous predictors. A multivariable general linear model was tested to examine the independent association of predictors with the composite outcome, which included anxiety/depression, PTSD symptoms, and work-related problems associated with perinatal loss. This model was used to calculate a risk score for each participant using a linear equation with the model parameter estimates as weights for each predictor. The resulting continuous risk score distribution was divided into quartiles, and the association of risk quartile with outcomes was examined using analysis of variance. SAS (Version 9.3, Cary, NC) was used for data analysis, with p<.05 considered significant.

## Results

Among 105 respondents, the mean age was 36 years with standard deviation of 11.2 years; 84 were female; nine were male; 61 were married; 43 were physicians; six were midwives; 35 were nurses or nurse practitioners; the remaining were medical technicians, clerks, and other staff (Table 1). Total possible number of participants is difficult to calculate for staff including clerical and technicians as the survey spanned over a period of months and across multiple departments throughout the hospital. It is suspected that total number of participants is approximately 230, making a response rate of about 46%. The average weekly hours worked was 34.4 with a standard deviation of 20.6 hours.

**Table 1:** Description of the survey respondents Response rates: overall estimated 46%, residents 51%, attendings estimated 64%, midwives estimated 75%. OB/GYN: obstetrician/ gynecologist; NICU: neonatal intensive care unit

Variable	N
Sex	
Female	84
Male	9
Other/Unknown	12
Religious	
Very strong	26
Somewhat strong	29
Not at all strong	19
Not affiliated	19
Unknown	12
Married	
Yes	61
No	15
Unknown	29
Patient care role	
Physician	43
- attending/ fellow	16
- resident	25
Midwife	6
Nurse	34
Nurse Practitioner	1
Medical Technician	2
Clerical	3
Other	4
Unknown	12
Type of Physician	
OB/GYN	37
Anesthesiology	6
Type of Nurse	
Labor & Delivery	19
Postpartum unit	9
NICU	5
Other	1
Reaction to losses	
Substance abuse	10
Mental health treatment	11
Career change	20

Six items measuring anxiety and depression symptoms were highly correlated, with Cronbach’s alpha being 0.87 and therefore combined into an anxiety/depression scale (Table 2) and referred to the two symptoms as anxiety/depression throughout this investigation. PTSD symptom items were highly correlated and were combined (Cronbach’s alpha 0.82, Table 2). Work-problem items had a high inter-item correlation and were combined (Cronbach’s alpha 0.84; Table 2). Four items measuring support at work following maternal or neonatal deaths were highly correlated and thus combined (Cronbach’s alpha 0.81; Table 2). The three predictor scales (anxiety/depression, PTSD symptoms, and work-related problems) were themselves highly correlated, so a second-order scale with their mean score was constructed (Cronbach’s alpha 0.81). This measured global negative repercussions following maternal/neonatal loss events.

**Table 2:** Item groups with high internal consistency reliability PHQ:  Patient Health Questionnaire;  PTSD: post-traumatic stress disorder

Construct	Cronbach’s alpha	Items	Item Wording
Anxiety/depression	.87	PHQ1	Feel nervous, anxious, on edge
		PHQ2	Not able to stop or control worrying
		PHQ3	Little interest or pleasure
		PHQ4	Feel down, depressed, hopeless
		Self-Blame	Self-blaming
		Isolated	Isolated
PTSD symptoms	.82	Intrusive symptoms	Nightmares about event or thought about it when you didn’t want to
		Avoidance	Try not to think about event; tried to avoid situations that reminded you of it
		Hypervigilance	Constantly on guard, watchful, easily startled
		Numb	Felt numb, detached from others, activities, your surroundings
		Relationship difficulties	Difficulties with interpersonal relationships
		Difficult going to work	Had difficulty going to work.
Work problems	.84	Decision making	Had a harder time making decisions at work.
		Patient interaction	My interactions with patients were negatively impacted.
		Colleague interaction	My interactions with colleagues were negatively impacted.
		Staff interaction	My interactions with other staff were negatively impacted
		Negative about work	Had negative feelings about going back to work.
Support at work	.81	Coworkers	I received support from my coworkers.
		Coworkers not	My coworkers were not supportive. (reversed)
		In charge	I received support from people in charge.
		In charge not	People in charge were not supportive. (reversed)
Second-order negative repercussion scale	.81	Anxiety/ Depression scale	
		PTSD scale	
		Work problem scale	

Univariate association with outcome

Provider variables significantly associated with negative repercussions included patient-care role (midwives had lowest negative repercussion scores, nurse practitioners, and medical technicians the highest), time since maternal death (highest negative repercussion score with more recent event), substance use (including but not limited to alcohol, drugs, tobacco), considering career change and seeking mental health treatment (Table 3). Religion and marital status did not have associations with negative repercussions (Table 3).

**Table 3:** Univariate association with global negative repercussions for all respondents *Statistically significant with p-value < .05

Predictor	Pearson r or mean (SD)	p-value
Age	r = -0.04	0.73
Sex		0.43
Male	-0.01 (0.52)	
Female	0.03 (0.65)	
Other/ Unknown	-0.57 (0.32)	
Religion		0.15
Very strong	0.15 (0.62)	
Somewhat strong	0.11 (0.63)	
Not at all strong	0.04 (0.54)	
Not affiliated	-0.36 (0.67)	
Unknown	-0.13 0.94)	
Married		0.76
Yes	0.02 (0.64)	
No	-0.03 (0.63)	
Weekly hours worked	r = -0.02	0.89
Patient-care role		0.028*
Physician	0.00 (0.54)	
Midwife	-0.54 (0.62)	
Nurse	0.05 (0.70)	
Nurse Practitioner	1.07 (n/a)	
Medical Technician	0.92 (0.11)	
N/A	-0.72 (0.10)	
Number of live births	r = -0.02	0.91
Involved in care of maternal death		0.24
Yes	-0.21 (0.71)	
No	0.07 (0.61)	
Unknown	0.06 (1.21)	
Level of involvement with maternal death		0.79
Very	-0.14 (0.65)	
Somewhat	-0.33 (0.79)	
Not very	-0.28 (0.96)	
On unit	0.44 (n/a)	
Time since care of maternal death (years ago)		0.0013*
0-1	0.19 (0.48)	
2-5	-0.41 (0.60)	
6-10	-1.37 (0.11)	
>10	0.13 (0.56)	
Involved in care of fetal/newborn death		0.83
Yes	-0.01 (0.66)	
No	-0.08 (0.75)	
Level of involvement with fetal / neonatal loss		0.16
Very	-0.08 (0.64)	
Somewhat	0.16 (0.70)	
Not very	0.47 (0.60)	
Time since care of fetal loss		0.11
0-1	0.05 (0.65)	
2-5	-0.22 (0.57)	
6-10	-0.32 (0.87)	
>10 years	0.56 (0.47)	
Unknown	-0.79 (n/a)	
Number of maternal or fetal deaths		0.24
1	0.09 (0.64)	
2-5	0.10 (0.60)	
6-9	-0.32 (0.56)	
10+	-0.12 (0.80)	
Unknown	0.57 (n/a)	
Substance use		0.003*
Yes	-0.54 (0.61)	
No	0.09 (0.60)	
Considered modifying career as a result		<0.0001*
Yes	-0.60 (0.47)	
No	0.22 (0.54)	
Sought mental health treatment		<0.0001*
Yes	-0.69 (0.32)	
No	0.12 (0.60)	
Is there a process to help providers?		0.07
Yes	-0.05 (0.62)	
No	-0.19 (0.63)	
Unknown	0.21 (0.62)	
Formal process is needed		0.22
Yes	-0.01 (0.65)	
No	0.38 (0.51)	
Unknown	-0.43 (0.51)	
Know whom to contact at workplace after a loss?		0.93
Yes	0.00 (0.60)	
No	0.01 (0.70)	

Multivariable model

**Figure 1:**
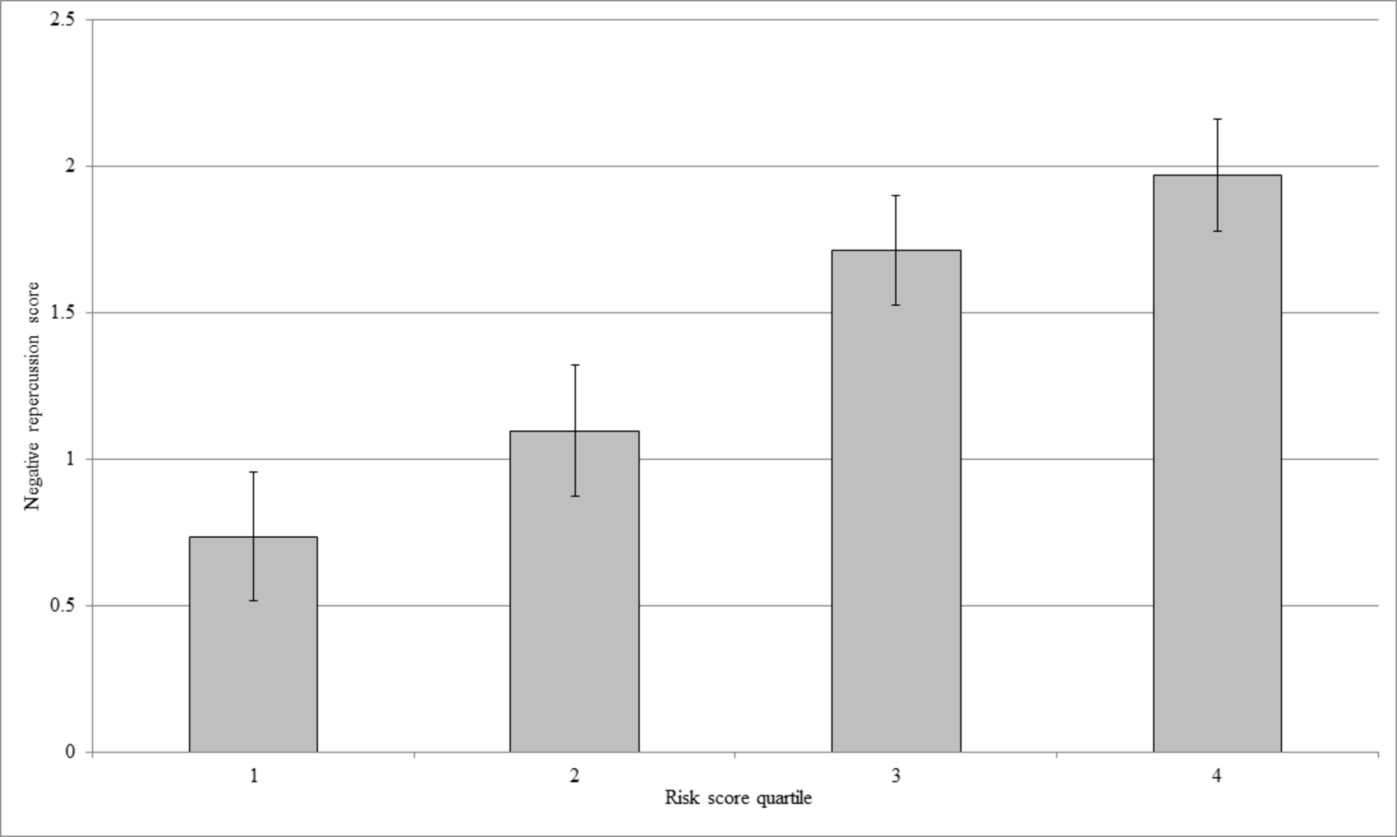
Association of risk score quartile with negative repercussion score The association between risk quartile and negative repercussion score was significant (p<.0001), and risk quartile explained 50% of the variance in negative repercussions (i.e. a strong effect).

Being a physician was protective after adjusting for other covariates (Table 4). Variables that also had significant independent associations with negative repercussions were substance use, considering a career change and seeking mental health treatment. All were negatively associated with the composite outcome. Based on the regression model parameters, a risk score was calculated for each participant using the equation Risk=0.226*non-physician - 0.487*substances - 0.654*Career Modification - 0.593*therapy, where each predictor was coded 1 if true, 0 if false (Figure 1). There was a strong association between risk score quartile and negative repercussion (R2=.50; p<.0001), indicating that the risk quartile may be useful as an indicator of providers likely to have high levels of anxiety/depression, PTSD symptoms, and work problems.

**Table 4:** Multivariate model predicting negative repercussion The equation to calculate risk for Negative Repercussion was: Risk=0.226*non-physician – 0.487*substances – 0.654*Career Modification – 0.593*therapy

Predictor	Parameter estimate (SE)	p-value
Patient role: non-physician	0.23 (0.11)	0.045
Substance use	-0.49 (0.16)	0.0036
Career modification	-0.65 (0.14)	<0.0001
Mental health treatment	-0.59 (0.16)	0.0005

Residents versus faculty

 A total of 49 residents were invited to complete the survey. Of residents invited (31 OB/GYN, 16 anesthesia), 25 completed the survey making a 51% response rate and 24 experienced an adverse event. Nine experienced a maternal death, while 22 experienced a perinatal death (Table 5). Most (83%) residents agreed that co-workers were supportive. Most (62%) agreed that the people in charge were supportive; 12.5% disagreed. Only 29% of residents had negative feelings about going back to work. One resident reported that their interactions with patients were negatively impacted although 16% agreed that they had a harder time making decisions.

**Table 5:** Physician involvement in maternal and perinatal deaths

Outcome	Resident Response	Attending Response
Experienced maternal death	36% (9/25)	25% (4/16)
- Somewhat/ very involved in maternal death	89% (8/9)	100% (4/4)
Experienced perinatal death	88% (22/25)	81% (13/16)
- Somewhat/ very involved in perinatal death	95% (21/22)	100% (13/13)
Experienced 2-5 maternal/ perinatal deaths	70.8% (17/24)	36% (5/14)

Almost half (48%) of the residents were aware of a process following an adverse event for providers and staff; most (80%) knew whom to contact for support following an event. Residents were more likely to be aware of support resources compared to general respondents. Only 54% of total respondents of the survey knew with whom to speak following an event. Both the majority of residents (92%) and all respondents (92%) think that a formal process is needed to address the consequences of an adverse event.

Table 6 and Table 7 discuss resident results in questions related to PTSD symptoms and depression screening. Three (12.5%) of the residents screened positive for PTSD symptoms; eight were positive for depression; three were positive for both. One resident (who did not screen positive for PTSD symptoms or depression) considered changing their career. Two sought mental health treatment, and two used substances.

**Table 6:** PHQ-4 responses from residents (out of 24 respondents), n(%) PHQ: Patient Health Questionnaire

	Response possibilities				
	Nearly every day	More than half the days	Several days	Not at all	
Feeling nervous, anxious or on edge	0 (0)	4 (16.7)	10 (41.7)	10 (41.7)	
Not being able to stop or control worrying	0 (0)	1 (4.2)	10 (41.7)	13 (54.2)	
Little interest or pleasure in doing things	0 (0)	1 (4.2)	7 (29.2)	16 (66.7)	
Feeling down, depressed or hopeless	0 (0)	2 (8.3)	10 (41.7)	12 (50.0)	
	Strongly agree	Agree	Neutral	Disagree	Strongly disagree
Self-blaming	2 (8.3)	5 (20.8)	3 (12.5)	8 (33.3)	6 (25.0)

**Table 7:** PC-PTSD responses from residents (out of 24 respondents) n(%) PC-PTSD: Primary Care Post-Traumatic Stress Disorder Screen

	Response possibilities				
	Yes	No			
Have had nightmares about the event or thought about it when you did not want to	9 (37.5)	15 (62.5)			
Tried hard not to think about the event or went out of your way to avoid situations that reminded you of the event	8 (33.3)	16 (66.7)			
Were constantly on guard, watchful or easily startled	3 (12.5)	21 (87.5)			
Felt numb or detached from others, activities or your surrounding	5 (20.8)	19 (79.2)			
	Strongly agree	Agree	Neutral	Disagree	Strongly disagree
Had difficulties with interpersonal relationships	0 (0)	5 (20.8)	3 (12.5)	5 (20.8)	11 (45.8)
Had difficulty going to work	0 (0)	6 (25.0)	4 (16.7)	8 (33.3)	6 (25.)

Compared to faculty, residents had a marginally higher negative overall outcome score (p=.07). Residents scored significantly higher than faculty on anxiety/depression (p=.03). There was no significant difference in levels of PTSD symptoms, work-related problems, or perceived support between residents and faculty.

**Figure 2:**
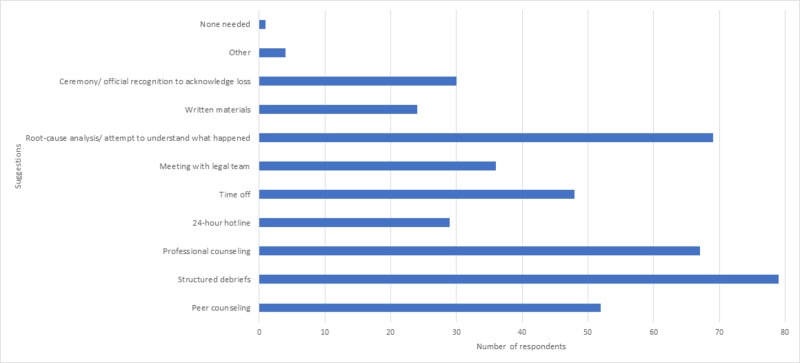
Suggestions for help following a maternal or fetal loss Responders were able to check all that apply

Suggestions for what should be included in a program after an adverse event include structured debriefs, root-cause analysis, professional counseling, peer counseling, and time off. (Figure 2)

## Discussion

Main findings

This is a single-institution observational study evaluating the impact of adverse perinatal/ neonatal and maternal events on obstetrical/maternal providers and staff. This appears to be the only study that focuses specifically on obstetrical adverse events.

Literature in other medical fields shows that the responses to patient death are similar to those found in this study: sadness, lack of sleep, exhaustion, crying, guilt, helplessness, depression [8, 10-11]. Some described feelings of relief if a patient had a long “dying trajectory” [10]. In contrast, relief would be an unusual response to adverse obstetrical events as these are usually unanticipated and occur quickly, leaving minimal time for prolonged “end-of-life” care.

This study demonstrates that it may be possible to predict which providers/staff are more likely to experience anxiety/depression and PTSD symptoms after a maternal or neonatal loss. The providers who are non-physicians, consider a career change, cope using substances, and seek mental health services are most likely to have negative repercussions. While institutions should provide support for all staff following an adverse event, they should particularly target those who meet these criteria. The risk quartile may be useful as an indicator of providers/staff who are likely to have high levels of anxiety, depression, PTSD symptoms, and work-related problems.

Most residents and faculty physicians who were involved with a maternal or fetal death described themselves as somewhat/very involved in that patient’s care. This data shows that residents had statistically significantly higher scores in anxiety/depression after maternal or neonatal death. They trended towards having a higher negative repercussion score overall, but the rates of work-related problems, PTSD symptoms, and receiving support in the workplace were similar to that of faculty physicians. Residents were significantly more impacted than faculty by adverse obstetrical events. With this information, program directors should ensure additional support to residents is offered.

Previous studies have shown that having a similarly aged patient to a provider or provider’s family member can influence the intensity of the experience [8, 11]. The responding resident ages range from 26 to 35, similar to reproductive-aged women, suggesting a possible explanation for greater vulnerability of residents compared to faculty. Not addressing the emotional impact of death on residents can have long-term effects on their individual lives and patient care, underscoring the importance of addressing the emotional component of these deaths [5, 11].

Previous publications have described a general lack of formal support or feedback from institutions following an adverse maternal/perinatal event [12]. Studies analyzing other specialties describe interventions following adverse events, but not all demonstrate that programs are helpful [3, 6-7, 13-15]. At GWUH, structured debriefing and peer counseling sessions exist, but effectiveness has not yet been evaluated.

Interestingly, much of the second-victim literature is written by non-physicians [8, 16-17]. It is notable that second victims are also nurses and other hospital personnel. The low response rate among these groups decreased the ability to comment more specifically.

Strengths and limitations

Study strengths include this being a novel, interdisciplinary population of obstetrics and maternity unit staff with a diverse patient population and provider demographics. Validated questionnaires for anxiety/depression and PTSD symptom screening were used. Study weaknesses include this being a single-center study and perhaps not generalizable to all women’s services providers. This survey was distributed at a single timepoint, independent of when adverse events occurred, possibly impacting a respondent’s recall of an event. The response rate from staff employees was low, leaving us unable to describe experiences, which may be unique to that group. Also, there was difficulty attaining the exact number of total possible participants in the setting of the diverse population attempted to incorporate, estimating about 230 potential participants, making the overall response rate just under 50%. Specifically, the resident response rate was 51%. It would be beneficial to develop a questionnaire administered post-event, which could place respondents into a risk quartile to help target at-risk individuals for intervention.

## Conclusions

Most care providers and staff at GWUH Women’s Services Department experience a maternal/perinatal loss. Anxiety/depression symptoms are common. Non-physicians and those who consider a career change, cope by using substances or seek mental health services are at higher risk for experiencing a negative repercussion. Residents are more likely to experience symptoms compared to attendings. Although systems are in place following an adverse event, staff may not be aware of how to use them. More data are needed in obstetrics to examine the developed interventions and to target those at increased risk for negative repercussions.

## References

[1] (2018). Pregnancy-Related Deaths. https://www.cdc.gov/reproductivehealth/maternalinfanthealth/pregnancy-relatedmortality.htm..

[2] MacDorman MF, Gregory EC (2015). Fetal and perinatal mortality: United States, 2013. Natl Vital Stat Rep.

[3] Deringer E, Caligor E (2014). Supervision and responses of psychiatry residents to adverse patient events. Acad Psychiatry.

[4] Harrison J, Evan E, Hughes A, Yazdani S, Federman M, Harrison R (2014). Understanding communication among health care professionals regarding death and dying in pediatrics. Palliat Support Care.

[5] Granek L, Tozer R, Mazzotta P, Ramjaun A, Krzyzanowska M (2012). Nature and impact of grief over patient loss on oncologists' personal and professional lives. Arch Intern Med.

[6] Bateman ST, Dixon R, Trozzi M (2012). The wrap-up: a unique forum to support pediatric residents when faced with the death of a child. J Palliat Med.

[7] Eagle S, Creel A, Alexandrov A (2012). The effect of facilitated peer support sessions on burnout and grief management among health care providers in pediatric intensive care units: a pilot study. J Palliat Med.

[8] Scott SD, Hirschinger LE, Cox KR, McCoig M, Brandt J, Hall LW (2009). The natural history of recovery for the healthcare provider "second victim" after adverse patient events. Qual Saf Health Care.

[9] Winkel AF, Nguyen AT, Morgan HK, Valantsevich D, Woodland MB (2017). Whose problem is it? The priority of physician wellness in residency training. J Surg Educ.

[10] Granek L, Bartels U, Scheinemann K, Labrecque M, Barrera M (2015). Grief reactions and impact of patient death on pediatric oncologists. Pediatr Blood Cancer.

[11] Meier DE, Back AL, Morrison RS (2001). The inner life of physicians and care of the seriously ill. JAMA.

[12] Harrison R, Lawton R, Stewart K (2014). Doctors' experiences of adverse events in secondary care: the professional and personal impact. Clin Med (Lond).

[13] Prabhakar D, Balon R, Anzia JM, Gabbard GO (2014). Helping psychiatry residents cope with patient suicide. Acad Psychiatry.

[14] Eng J, Schulman E, Jhanwar SM, Shah MK (2015). Patient death debriefing sessions to support residents' emotional reactions to patient deaths. J Grad Med Educ.

[15] Jefee-Bahloul H, Hanna RC, Brenner AM (2014). Teaching psychiatry residents about suicide loss: impact of an educational program. Acad Psychiatry.

[16] Scott SD, Hirschinger LE, Cox KR (2010). Caring for our own: deploying a systemwide second victim rapid response team. Jt Comm J Qual Patient Saf.

[17] Wu AW (2000). Medical error: the second victim. The doctor who makes the mistake needs help too. BMJ.

